# Application of *in vivo *micro-computed tomography in the temporal characterisation of subchondral bone architecture in a rat model of low-dose monosodium iodoacetate-induced osteoarthritis

**DOI:** 10.1186/ar3543

**Published:** 2011-12-21

**Authors:** Geetha Mohan, Egon Perilli, Julia S Kuliwaba, Julia M Humphries, Ian H Parkinson, Nicola L Fazzalari

**Affiliations:** 1 Bone and Joint Research Laboratory, Directorate of Surgical Pathology, SA Pathology and Hanson Institute, Frome Road, Adelaide, SA 5000, Australia; 2 Discipline of Anatomy and Pathology, School of Medical Sciences, The University of Adelaide, Adelaide, SA 5005, Australia

## Abstract

**Introduction:**

Osteoarthritis (OA) is a complex, multifactorial joint disease affecting both the cartilage and the subchondral bone. Animal models of OA aid in the understanding of the pathogenesis of OA and testing suitable drugs for OA treatment. In this study we characterized the temporal changes in the tibial subchondral bone architecture in a rat model of low-dose monosodium iodoacetate (MIA)-induced OA using *in vivo *micro-computed tomography (CT).

**Methods:**

Male Wistar rats received a single intra-articular injection of low-dose MIA (0.2 mg) in the right knee joint and sterile saline in the left knee joint. The animals were scanned *in vivo *by micro-CT at two, six, and ten weeks post-injection, analogous to early, intermediate, and advanced stages of OA, to assess architectural changes in the tibial subchondral bone. The articular cartilage changes in the tibiae were assessed macroscopically and histologically at ten weeks post-injection.

**Results:**

Interestingly, tibiae of the MIA-injected knees showed significant bone loss at two weeks, followed by increased trabecular thickness and separation at six and ten weeks. The trabecular number was decreased at all time points compared to control tibiae. The tibial subchondral plate thickness of the MIA-injected knee was increased at two and six weeks and the plate porosity was increased at all time points compared to control. At ten weeks, histology revealed loss of proteoglycans, chondrocyte necrosis, chondrocyte clusters, cartilage fibrillation, and delamination in the MIA-injected tibiae, whereas the control tibiae showed no changes. Micro-CT images and histology showed the presence of subchondral bone sclerosis, cysts, and osteophytes.

**Conclusions:**

These findings demonstrate that the low-dose MIA rat model closely mimics the pathological features of progressive human OA. The low-dose MIA rat model is therefore suitable to study the effect of therapeutic drugs on cartilage and bone in a non-trauma model of OA. *In vivo *micro-CT is a non-destructive imaging technique that can track structural changes in the tibial subchondral bone in this animal model, and could also be used to track changes in bone in preclinical drug intervention studies for OA treatments.

## Introduction

Osteoarthritis (OA) is generally a slow progressive joint disease characterized by loss of articular cartilage, subchondral bone sclerosis, cysts, and osteophyte formation [1]. The etiopathology of OA remains obscure and currently there are no pharmacological interventions available to halt or reverse the progression of OA. Animal models of OA are of considerable importance as they are not only useful to study the pathogenesis and progression of OA, but also to evaluate suitable therapeutic drugs for OA treatment. Moreover, knowledge of early pathological changes is essential for early treatment options and to develop better therapeutic agents to modify the disease progression.

The monosodium iodoacetate (MIA)-induced OA rat model is a minimally invasive animal model that reproduces cartilage and bone pathology similar to human OA [2]. The onset, progression and severity of OA can be easily controlled in this model by changing the dose of MIA, which makes it useful to study disease progression and the effect of disease modifying osteoarthritis drugs (DMOAD). A dose response study by Guingamp *et al. *showed that the severity of cartilage degradation depends on the dosage of MIA injected into the knee joint. Higher doses of MIA (up to 3 mg) caused cartilage erosion, sclerosis and exposure of subchondral bone on day 15 post MIA injection, and on day 30 there was complete loss of articular cartilage, with greatly remodelled subchondral bone [3], whereas, a low-dose of MIA (0.25 mg) induced moderate cartilage damage at 3 weeks [4]. In a pilot study, we evaluated the dose responsiveness of tibial cartilage and subchondral bone to MIA using a high-dose of 2 mg MIA (*n *= 3) and a low-dose of 0.2 mg MIA (*n *= 3) in rats. As early as after two weeks, high-dose MIA induced characteristic features of end-stage human OA such as loss of tibial articular cartilage, exposure of subchondral bone, subchondral trabecular bone erosion, cysts and osteophytes. In contrast, these changes were observed only at ten weeks in the low-dose MIA model (Mohan G *et al*: unpublished observations). The low-dose MIA model, of relatively slow progressing OA, enables *in vivo *monitoring of tissue-level changes representative of progressive human OA; whereas, in the high-dose model the disease progression is very rapid, which is less suitable for longitudinal monitoring of cartilage and subchondral bone changes.

The tissue-level characterization of animal models of OA has traditionally been done by histological assessment. Developments in X-ray micro-computed tomography (micro-CT) allow *in vivo *imaging of small animal models, with high spatial resolution and at subsequent time points, to study bone architecture [5-7]. This imaging technique enables non-destructive assessment of the bone both quantitatively and qualitatively. Moreover, the use of a longitudinal study design using *in vivo *micro-CT allows the quantification of subchondral bone in the same animal over time. Recent studies have used micro-CT to quantify structural changes in the tibial subchondral bone in the MIA rat model; however, those studies have utilized high-dose MIA injections, with the rats examined only at the end point of the studies [8-10]. The temporal changes in the structure of subchondral trabecular bone during the progression of OA in the low-dose MIA model have not been previously described.

The purpose of this study was to characterize the structural changes in the tibial subchondral bone using longitudinal *in vivo *micro-CT imaging in an animal model that closely mimics human progressive OA. It is hypothesised that low-dose MIA will induce cartilage changes and measurable, progressive tibial subchondral bone changes at two, six, and ten weeks post-injection.

## Materials and methods

### Animals and OA induction

Twelve 8-week-old male Wistar rats (Animal Resource Center, Canning Vale, WA, Australia), weighing 200 - 230 g at the start of the experiment, were used. They were kept in a sanitary ventilated animal room with controlled temperature (20 to 24°C), a light-dark cycle (12 hour/12 hour) and were fed standard laboratory rodent chow (Specialty Feeds, Glen Forrest, WA, Australia) with water available *ad libitum*. The rats were acclimatized for one week before the start of the experiment. They were closely monitored and daily clinical record sheets were completed throughout the duration of the study by the animal care facility staff.

On day 0, the rats were anesthetized with an isoflurane/O_2 _mixture and both their knees were shaved and disinfected with 70% alcohol. Each rat was given a single intra-articular injection of 0.2 mg MIA through the infrapatellar ligament of the right knee. MIA was dissolved in sterile physiologic saline and administered in a volume of 50 μl using a 26-gauge 0.5-inch needle. The left contralateral control knee was injected with 50 μl of sterile physiologic saline.

The hind limbs of all the rats were imaged with high-resolution *in vivo *micro-CT at two, six, and ten weeks post-MIA injection, which represent early, intermediate, and advanced stages of OA. Double-fluorescent labelling of newly formed bone was achieved by using calcein (5 mg/kg body weight; Sigma-Aldrich, Sydney, NSW, Australia) administered intraperitoneally on day 7 before sacrifice, followed by xylenol orange (90 mg/kg body weight; Sigma-Aldrich, Australia) administered intraperitoneally 2 days before sacrifice. The animal handling and experimental procedures outlined in this study were carried out in accordance with The University of Adelaide Animal Ethics Committee and The Institute of Medical and Veterinary Science Animal Ethics Committee.

### *In vivo *micro-CT imaging

**Figure 1:**
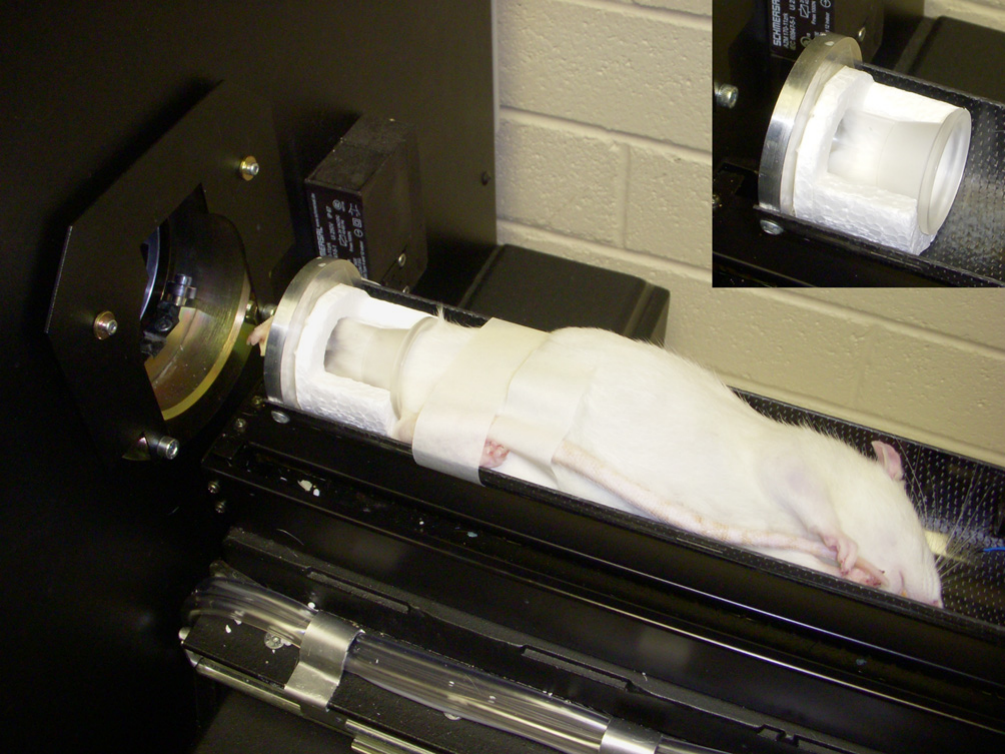
***In vivo *micro-CT imaging of a rat hind limb; anaesthetized rat on the scanner bed in supine position, with the hind limb secured in a customised leg fixative device (inset)**. Micro-CT, micro-computed tomography.

*In vivo *micro-CT imaging was performed using a bench-top cone-beam type *in vivo *animal scanner (Skyscan model 1076, Skyscan, Kontich, Belgium) [6]. Each rat was anesthetized with isofluroane/O_2 _and placed in the scanner bed in a supine position (Figure 1). The MIA-injected knees and the contralateral control knees were scanned separately. During each scan only the knee for image data acquisition was irradiated, while the contralateral limb and the rest of the body were lead-shielded from radiation. The hind limb of the rat was secured into a customized leg fixative device consisting of a cylindrical plastic holder fitted to a polystyrene tube. This allowed positioning of the hind limb close to the central scanner axis, preventing any movement of the limb during scanning. The total scan time for each limb was 20 minutes during which the rat was under anesthesia. The scans were performed using the following scanner settings: X-ray source voltage 60 kVp, current 100 μA, a 1-mm thick aluminum filter to reduce beam-hardening artefact, 1 frame averaging. The pixel size was 8.7 μm, the exposure time was 4.7 seconds, and the rotation step was 0.8°, with a complete rotation over 197° [6]. The cross-sectional images were reconstructed using a filtered back-projection algorithm (NRecon, V 1.4.4, Skyscan, Kontich, Belgium). For each scan, a stack of 1,800 cross-sections was reconstructed, centered over the knee-joint (total reconstructed height about 16 mm), with an interslice distance of 1 pixel (8.7 μm). The reconstructed images were of 1,500 × 1,500 pixels each, 8.7 μm pixel size, and were stored as 8-bit images (256 grey levels) [6].

### Bone histomorphometric analysis

#### Subchondral trabecular bone

**Figure 2:**
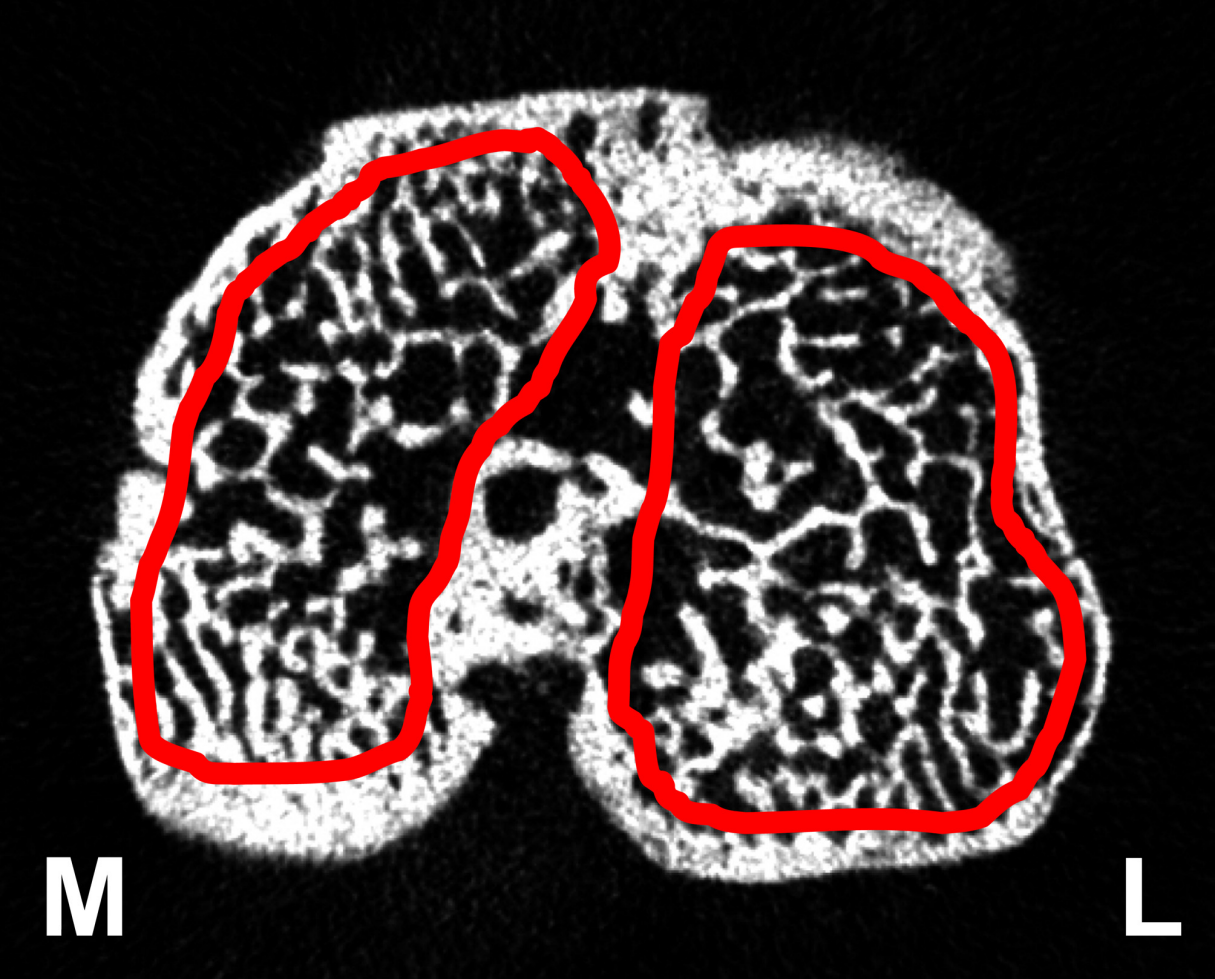
**Axial micro-CT image of a rat tibia, with the region of interest (solid red line) in the medial (M) and lateral (L) subchondral bone compartment**. Micro-CT, micro-computed tomography.

On the stack of the reconstructed micro-CT cross-section images, manual regions of interest (ROI) of an irregular anatomical contour were drawn in the subchondral trabecular bone region for the medial and lateral tibial plateau (Figure 2), for both the MIA-injected knee and the control knee (software CT Analyser, V 1.8.05, Skyscan, Kontich, Belgium). The volume of interest (VOI) consisted of a stack of ROIs drawn over 52 cross-sections, resulting in a height of 0.45 mm. The VOI included the subchondral trabecular bone starting below the subchondral plate, and extending distally towards the growth plate, excluding both the cortical bone and growth plate interface. The VOI used was of the same size and shape for all the three time points (two, six and ten weeks), for both the control and the MIA-injected tibia. For the calculation of the morphometric parameters, the images were segmented using a uniform threshold as described in a previously published paper [6]. The following three-dimensional (3D) morphometric parameters were calculated for the medial VOI, the lateral VOI and the total (= medial + lateral) VOI of subchondral trabecular bone (software CT Analyzer, Skyscan): bone volume (BV, mm^3^), bone volume fraction (BV/TV, %), trabecular thickness (Tb.Th, μm), trabecular separation (Tb.Sp, μm) and trabecular number (Tb.N, 1/mm). BV is the volume in 3D of the structure segmented as bone, and BV/TV is the ratio of the segmented bone volume to the total volume of the region of interest. Tb.Th is the mean thickness of the trabeculae, Tb.Sp is the mean distance between trabeculae, Tb.N is the average number of trabeculae present per unit length. Tb.Th and Tb.Sp were assessed using direct 3D methods, Tb.N was calculated using the formula Tb.N = (BV/TV)/Tb.Th [6,11,12].

#### Subchondral plate

In the binarized images, the subchondral plate was separated from the trabecular bone of the tibia by using a software kindly provided by Botter *et al. *[13]. The 3D subchondral plate thickness (Pl.Th, μm) and porosity (volume of the pores in the plate over the total volume of the plate, Pl.Por, %) of the medial and lateral compartments of the tibial subchondral plate were calculated on regions measuring 1.5 mm medio-lateral in width, 2.5 mm in length from the posterior side. For both parameters (Pl.Th and Pl.Por), the data from the medial and lateral compartments were averaged to obtain the value of the total compartment [13].

### Macroscopic analysis

At ten weeks post-MIA injection the rats were euthanized with a CO_2 _overdose and both the right and left tibiae were dissected for macroscopic study. Soft tissues surrounding the tibia were carefully removed to prevent articular cartilage damage. The image of the tibial cartilage was recorded using a fluorescence stereomicroscope (SZX 10, Olympus). With the microscope, the fluorochrome labels calcein and xylenol orange were imaged and recorded. The macroscopic lesions were graded as follows: 0 = normal appearance, 1 = slight yellowish discoloration of the chondral surface, 2 = little cartilage erosions in load-bearing areas, 3 = large erosions extending down to the subchondral bone, and 4 = large erosion with large areas of subchondral bone exposure [3]. Two blinded observers graded the medial and lateral tibial plateaus separately. The scores for the tibiae were combined and the average values were determined for the MIA-injected knee and the control knee.

### Histological analysis

After imaging, the tibiae (*n *= 6 rats; tibiae from the other 6 rats were used in another analysis) were fixed in 4% paraformaldehyde, decalcified and embedded in paraffin. Three coronal sections (5 μm thick) 100 μm apart were obtained and stained with 1.5% Safranin O, and 0.02% fast green counter stain. The sections were observed for OA-like features such as surface discontinuity, loss of proteoglycans, disorientation of chondrocytes, subchondral bone sclerosis and presence of cysts and osteophytes.

The Osteoarthritis Research Society International (OARSI) scoring method [14] was used to grade and stage tibial cartilage in the MIA-injected knee and control knee. For each histological slide, the OA score was obtained by multiplying the grade and stage values. The OA score ranges from 0 (representing no OA activity) to 24 (highest OA degradation).

### Serum C-reactive protein (CRP) analysis

Blood samples were obtained by tail venipunture from the MIA-injected rats (*n *= 6) and a group of age-matched control rats (*n *= 4, from another non-micro-CT imaging study) under isofluroane anaesthesia. Blood samples were obtained from the rats at pre-injection, two, six, and ten week time points. Serum samples were collected and stored at -80°C until analyzed. Serum levels of CRP, a marker of systemic inflammation, were measured by enzyme-linked immunosorbent assay according to the manufacturer's protocol (Rat CRP ELISA kit, BD Biosciences, San Diego, CA, USA).

### Statistical analysis

The morphometric parameters had a normal distribution (Shapiro-Wilk test, *P *> 0.05 for all the examined parameters). For each morphometric parameter it was determined whether there was (a) a 'time effect' and (b) a 'time by group interaction effect'. The 'time effect' indicates if there was any change over time within each group (tibia of control and MIA-injected knee) and the 'time by group interaction effect' indicates if the different groups showed different patterns of changes over time. For each tibia, 3D morphometric parameters and serum CRP data sets were examined using analysis of variance for repeated measures (two-way repeated measures ANOVA). If F-values for a given variable were found to be significant, a paired Student's t-test was applied to assess the changes in the morphometric parameters and CRP between the MIA-injected knee and control knee. The *P*-values were adjusted for repeated comparisons by Holm's Bonferroni stepdown procedure [15]. Similarly, a paired Student's t-test with *P*-values adjusted for repeated comparison by Holm's Bonferroni stepdown procedure was done to assess changes in each tibia between time points. The overall type I error rate was set at an alpha level of 0.05.

## Results

### Micro-CT

#### Bone histomorphometric changes

**Figure 3:**
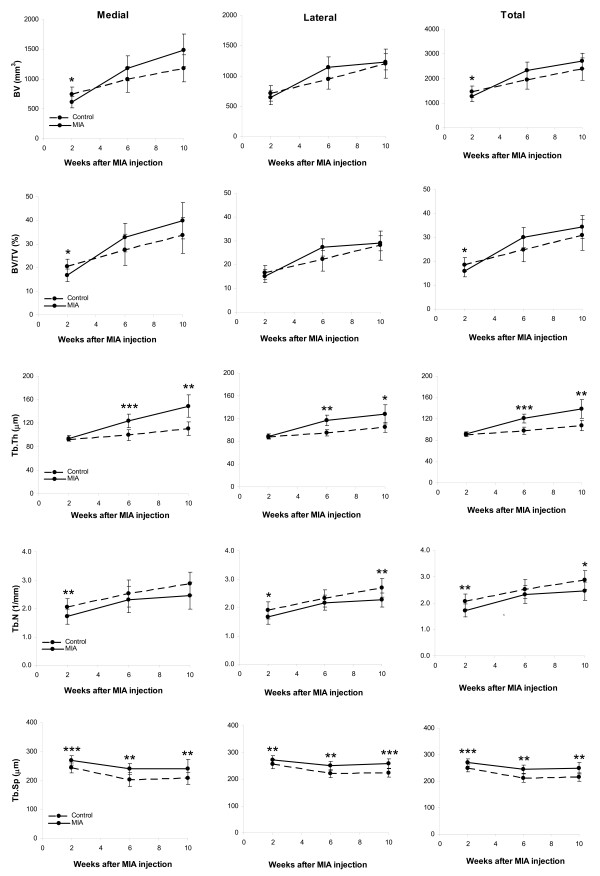
**Plots of morphometric parameters of subchondral trabecular bone determined by micro-CT in the control tibiae and in the MIA-injected tibiae, at two, six, and ten weeks post-MIA injection**. There was a statistically significant increase over time in BV, BV/TV, Tb.Th, and Tb.N, and a decrease in Tb.Sp, in both the control and MIA-injected tibiae. Error bars = SD. BV, bone volume; BV/TV, bone volume fraction; CT, computed tomography; MIA, monosodium iodoacetate; TB.N, trabecular number; Tb.Sp, trabecular separation; Tb.Th, trabecular thickness. * *P *< 0.05, ** *P *< 0.01, *** *P *< 0.001, between control tibiae and MIA-injected tibiae.

The 'time effect' was significant for all the parameters. The 'time by group interaction effect' was significant for BV, BV/TV, Tb.Th, Pl.Th and Pl.Por. There was a significant increase in total BV, BV/TV, Tb.Th, Tb.N, and Pl.Th. in the tibial subchondral bone of both the MIA-injected knee and the control knee over time (*P *< 0.05, Figure 3). This was due to the natural growth of the animal. The total Tb.Sp of both the control tibia and the tibia of the MIA-injected knee were significantly decreased at ten weeks compared to two weeks (*P *< 0.001, Figure 3).

#### Subchondral trabecular bone

Table 1 shows the summarized data and comparison of the tibial subchondral trabecular bone microarchitectural parameters of the MIA-injected knee and the control knee at two, six, and ten weeks post-MIA injection. At two weeks there was a significant decrease in BV and BV/TV in the MIA-injected tibia compared to the control tibia, in both the medial and the total compartments (-18% and -14%, respectively, *P *< 0.05 for both parameters). However, at six and at ten weeks post-MIA injection, although not statistically significant, there was an increase in tibial subchondral trabecular BV and BV/TV in the MIA-injected knee compared to the control knee, particularly in the medial compartments (19% for BV and BV/TV at 6 weeks and 26%, 19%, for BV and BV/TV at 10 weeks, respectively).

**Table 1:** Morphometric parameters of tibial subchondral trabecular bone in the MIA-injected tibiae and in the control tibiae, at 2, 6, and 10 weeks post-MIA injection

	2 Weeks				6 Weeks				10 Weeks			
	**OA**	**Control**	**%d**	**p-value**	**OA**	**Control**	**%d**	**p-value**	**OA**	**Control**	**%d**	**p-value**
	**Mean ± SD**	**Mean ± SD**	**OA-CTL**		**Mean ± SD**	**Mean ± SD**	**OA-CTL**		**Mean ± SD**	**Mean ± SD**	**OA-CTL**	

**BV (mm^3^)**												
Medial	613 ± 97	745 ± 118	-17.8	0.018	1177 ± 211	993 ± 214	18.6	0.102	1481 ± 271	1179 ± 229	25.6	0.102
Lateral	647 ± 120	711 ± 130	-9.0	0.116	1145 ± 168	948 ± 169	20.7	0.116	1229 ± 133	1205 ± 241	2.0	0.709
Total	1260 ± 202	1456 ± 239	-13.5	0.048	2322 ± 341	1941 ± 370	19.7	0.098	2710 ± 326	2384 ± 454	13.7	0.116
**BV/TV (%)**												
Medial	16.8 ± 2.6	20.4 ± 3.2	-18.0	0.018	32.7 ± 6.5	27.5 ± 6.6	19.1	0.156	39.8 ± 7.7	33.6 ± 7.6	18.5	0.156
Lateral	15.1 ± 2.8	16.7 ± 3.0	-9.1	0.156	27.2 ± 3.6	22.2 ± 3.8	22.5	0.156	29.0 ± 3.2	28.2 ± 6.0	3.0	0.592
Total	15.9 ± 2.5	18.6 ± 3.0	-14.0	0.032	30.0 ± 4.2	24.8 ± 5.0	20.6	0.154	34.4 ± 4.8	30.9 ± 6.4	11.5	0.156
**Tb.Th (μm)**												
Medial	94 ± 5	92 ± 3	2.1	0.396	124 ± 11	100 ± 9	24.6	p < 0.001	149 ± 19	110 ± 12	34.9	0.001
Lateral	89 ± 4	87 ± 3	1.6	0.360	117 ± 9	95 ± 5	23.1	0.001	128 ± 17	105 ± 8	22.0	0.012
Total	91 ± 4	90 ± 3	1.9	0.396	120 ± 9	97 ± 7	23.9	p < 0.001	138 ± 18	108 ± 10	28.6	0.002
**Tb.N (1/mm)**												
Medial	1.8 ± 0.3	2.2 ± 0.3	-19.9	0.009	2.5 ± 0.5	2.7 ± 0.5	-9.5	0.405	2.6 ± 0.5	3.1 ± 0.4	-13.7	0.112
Lateral	1.7 ± 0.3	1.9 ± 0.3	-12.2	0.020	2.2 ± 0.3	2.3 ± 0.3	-6.8	0.405	2.3 ± 0.3	2.7 ± 0.3	-16.0	0.009
Total	1.7 ± 0.2	2.1 ± 0.3	-16.3	0.009	2.3 ± 0.3	2.5 ± 0.4	-8.3	0.405	2.5 ± 0.3	2.9 ± 0.4	-14.8	0.012
**Tb.Sp (μm)**												
Medial	270 ± 16	243 ± 17	10.8	p < 0.001	240 ± 19	203 ± 24	17.9	0.002	241 ± 32	207 ± 20	16.3	0.005
Lateral	272 ± 16	256 ± 17	6.2	0.001	250 ± 16	221 ± 14	13.2	0.001	258 ± 17	223 ± 15	15.7	p < 0.001
Total	271 ± 13	250 ± 14	8.4	p < 0.001	245 ± 15	212 ± 16	15.5	0.001	245 ± 22	215 ± 15	13.8	0.001

Data represented as mean ± SD, % d is the percentage difference in values for tibial subchondral bone between the control and MIA-injected knee. BV, bone volume; BV/TV, bone volume fraction; MIA, monosodium iodoacetate; OA, osteoarthritis; OA-CTL, osteoarthritis control; SD, standard deviation; Tb.N, trabecular number; Tb.Sp, trabecular separation; Tb.Th, trabecular thickness.

At two weeks, no statistically significant difference was observed in Tb.Th in all the three tibial compartments between the MIA-injected knee and control. However, at six and ten weeks, there was a significant increase in Tb.Th in the MIA-injected knee compared to the control knee in the medial (25%, *P *< 0.001, and 35%, *P *< 0.01, respectively), lateral (23%, *P *< 0.01, and 22%, *P *< 0.05, respectively) and total (24%, *P *< 0.001, and 29%, *P *< 0.01, respectively) tibial compartments. The Tb.N was significantly lower in the MIA-injected knee compared to the control knee in all the three tibial compartments at two weeks and in the lateral and total compartments at ten weeks post-MIA injection. At 2 weeks, the Tb.N was reduced by 20% (*P *< 0.01), 12% (*P *< 0.05), and 16% (P < 0.01) in the medial, lateral and total compartments, respectively. At six weeks, the Tb.N did not differ significantly between the tibia of the MIA-injected knee and control knee. At 10 weeks, the Tb.N was decreased by 14%, 16% (*P *< 0.01) and 15% (*P *< 0.05) in the medial, lateral and total compartments, respectively, when compared to the control knee. The Tb.Sp in the MIA-injected knee was significantly higher than in the control knee at all the three time points, in all the three tibial compartments. At 2 weeks, the Tb.Sp of the MIA-injected knee was increased by 11% (*P *< 0.001), 6% (*P *< 0.01) and 8% (*P *< 0.001) in the medial, lateral and total compartments, compared to control. Similarly, at 6 weeks, in the MIA-injected knee there was an increase of 18%, 13% and 16% (*P *< 0.01 for all compartments), respectively, and at 10 weeks there was an increase of 16% (*P *< 0.01), 16% (*P *< 0.001) and 14% (*P *< 0.01), in the medial, lateral and total compartments, respectively, compared to control.

#### Subchondral plate

The tibial subchondral plate thickness of the MIA-injected knee increased significantly at two weeks (*P *< 0.01 for medial compartment) and six weeks (*P *< 0.01 for both medial and total compartments) post injection compared to the control knee. At ten weeks there was no significant difference in thickness (medial, lateral or total) between the MIA-injected knee and the control knee (Table 2). The porosity of the tibial subchondral plate was significantly increased in the MIA-injected knee compared to the control knee at all time points in all the three compartments analyzed (*P *< 0.01).

**Table 2:** Tibial subchondral plate thickness and porosity of the MIA-injected knee and control knee, at 2, 6, and 10 weeks post-MIA injection.

	Subchondral Plate Thickness, Pl.Th (μm)									Subchondral Plate Porosity, Pl.Por (%)					
	Medial			Lateral			Total			Medial		Lateral		Total	
										% d		% d		% d	
	OA	Control	p-value	OA	Control	p-value	OA	Control	p-value	OA - CTL	p-value	OA - CTL	p-value	OA - CTL	p-value
**2 weeks**	114 ± 7	105 ± 7	0.027	101 ± 6	109 ± 7	0.305	107 ± 4	107 ± 8	0.770	23	0.004	24	0.004	24	0.003
**6 weeks**	146 ± 32	119 ± 32	0.024	130 ± 17	120 ± 26	0.880	138 ± 23	118 ± 26	0.028	63	0.001	69	p < 0.001	66	p < 0.001
**10 weeks**	181 ± 27	175 ± 31	0.520	166 ± 18	178 ± 24	0.318	177 ± 19	177 ± 25	0.473	66	0.004	77	0.004	71	0.003

Data represented as mean ± SD for plate thickness, % d is the percentage difference in plate porosity between the control and MIA-injected knee. MIA, monosodium iodoacetate; OA, osteoarthritis; OA-CTL, osteoarthritis control; SD, standard deviation

#### Qualitative subchondral bone changes

**Figure 4:**
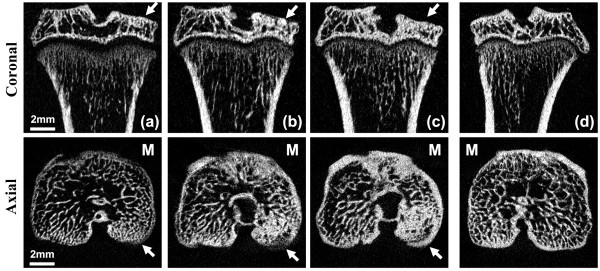
**Coronal and axial micro-CT images of an MIA-injected tibia at two weeks (a), six weeks (b) and ten weeks (c) post injection, together with a control tibia at ten weeks (d)**. The MIA-injected tibia showed altered subchondral bone architecture, with sclerosis on the medial tibial compartment (M) (indicated by arrow) at six and ten weeks after injection, whereas the control tibia showed no sclerosis at ten weeks. Micro-CT, micro-computed tomography; MIA, monosodium iodoacetate.

**Figure 5:**
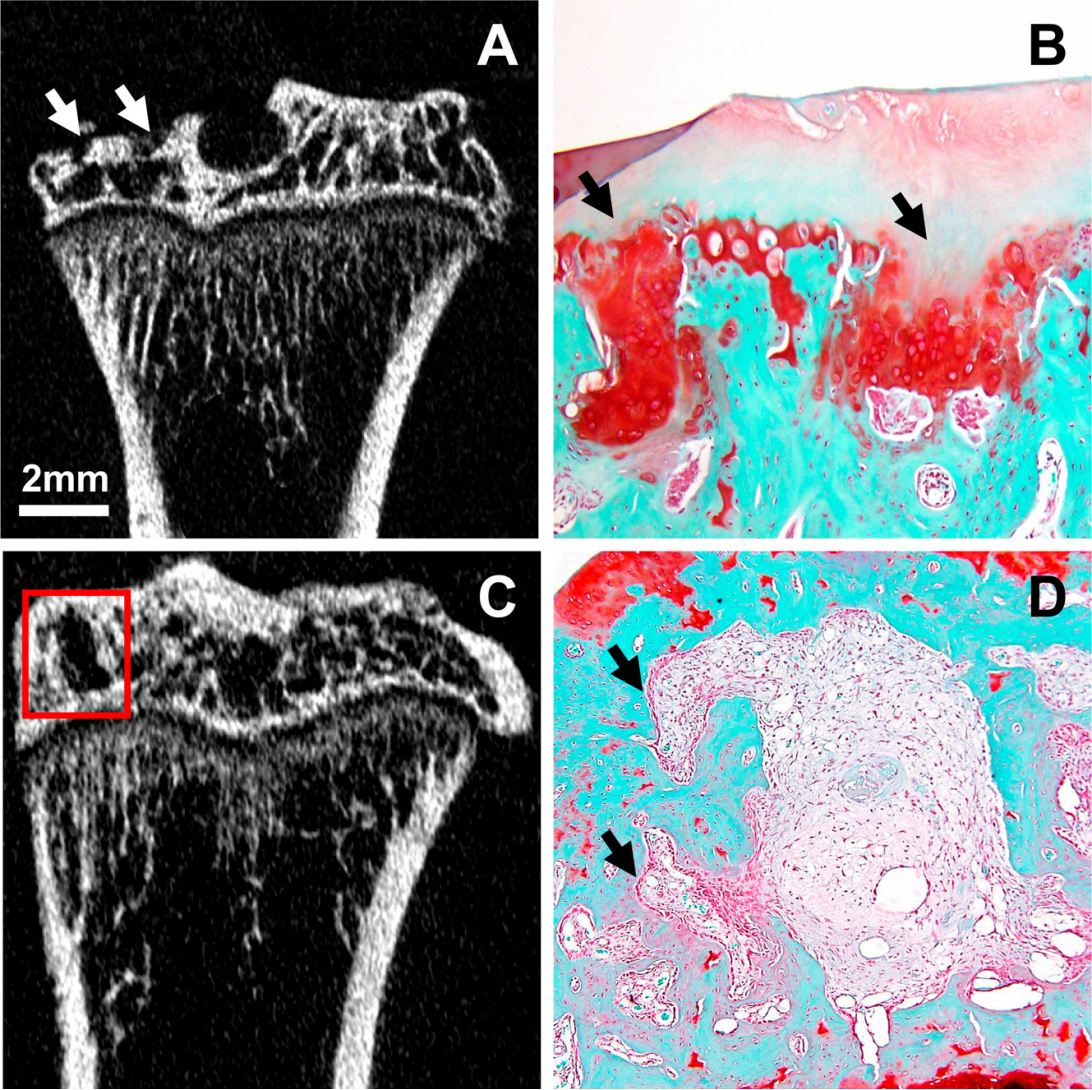
**Coronal micro-CT image (A) and histology section (B) of tibia from an MIA-injected knee, showing subchondral plate breach by the activated chondrocytes as a result of abnormal repair, as indicated by arrows (A, B)**. Micro-CT image revealed the presence of focal subchondral bone lesions in the medial tibial plateau **(C) **at ten weeks post injection (rectangle). The safranin O and fast green stained section of the MIA-injected knee at ten weeks post injection **(D) **confirmed the presence of subchondral bone cyst in the areas of bone lesions as predicted by micro-CT image. The cysts were found to be surrounded by sclerotic bone with fibrous tissue containing debris of necrotic bone. Note the presence of large active osteoblasts (arrow) lining the trabeculae adjacent to the sites of bone resorption. Original magnification × 100. Micro-CT, micro-computed tomography; MIA, monosodium iodoacetate.

**Figure 6:**
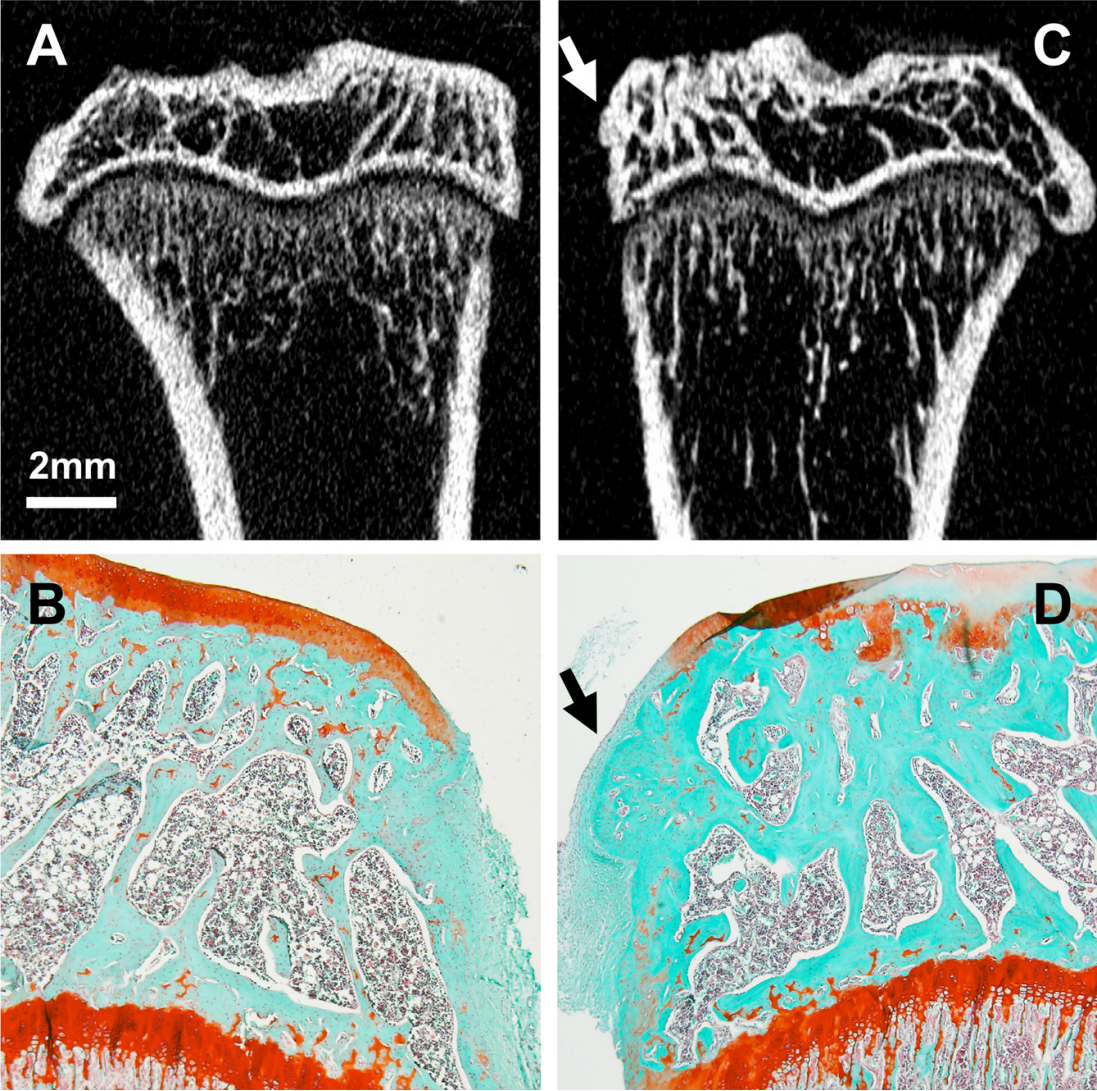
**Micro-CT coronal images of tibia from a control knee (A) and a MIA-injected knee (C) at ten weeks post injection**. Micro-CT images revealed the presence of marginal tibial osteophytes in all the rats in the MIA-injected knee (C, arrow), whereas the control knee injected with saline showed no osteophyte-like structure formation (A). The normal control tibia **(B) **and the marginal tibial osteophytes in the MIA-injected knee (**D**, arrow) were confirmed by histology at ten weeks post injection. The osteophytes contained marrow spaces filled with fibrous bone marrow cells. Original magnification × 40. Micro-CT, micro-computed tomography; MIA, monosodium iodoacetate.

**Figure 7:**
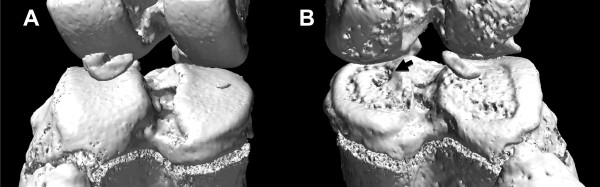
**Three-dimensional surface rendering obtained from micro-CT images of a control knee (A) and of a MIA-injected knee (B) at ten weeks after injection**. The control knee maintained the subchondral plate integrity with a smooth contour (A). The MIA-injected knee showed erosion and pitting of the tibial subchondral plate, which was more severe in the medial tibial plateau, as indicated by arrow (B). Micro-CT, micro-computed tomography; MIA, monosodium iodoacetate.

All the rats showed pathological subchondral bone changes in the MIA-injected knee, whereas the contralateral control knee showed no OA-like changes in the tibial subchondral bone microarchitecture throughout the study duration (ten weeks). Morphologic evaluation of the MIA-injected knee over time showed sclerosis in the tibial subchondral bone at six and ten weeks after MIA injection, particularly in the medial tibial condyle (Figure 4). At six and ten weeks, the tibial subchondral plate of the MIA-injected knee was breached in focal areas, these breaches were confirmed by histology (Figure 5A, B). From the micro-CT images, in 8 out of 12 rats (67%), empty spaces were observed in the medial tibial subchondral bone of the MIA-injected knee at 6 and 10 weeks. These empty spaces were confirmed as cysts from the histology sections (Figure 5C, D). Marginal osteophytes were observed in all the rats at six and ten weeks from the micro-CT images and these osteophytes were confirmed by histology (Figure 6). Three-dimensional surface rendering of the MIA-injected knee showed erosion and pitting of the tibial subchondral plate, which was more severe in the medial tibial plateau, whereas the control knee maintained the subchondral plate integrity (Figure 7).

### Macroscopy

**Figure 8:**
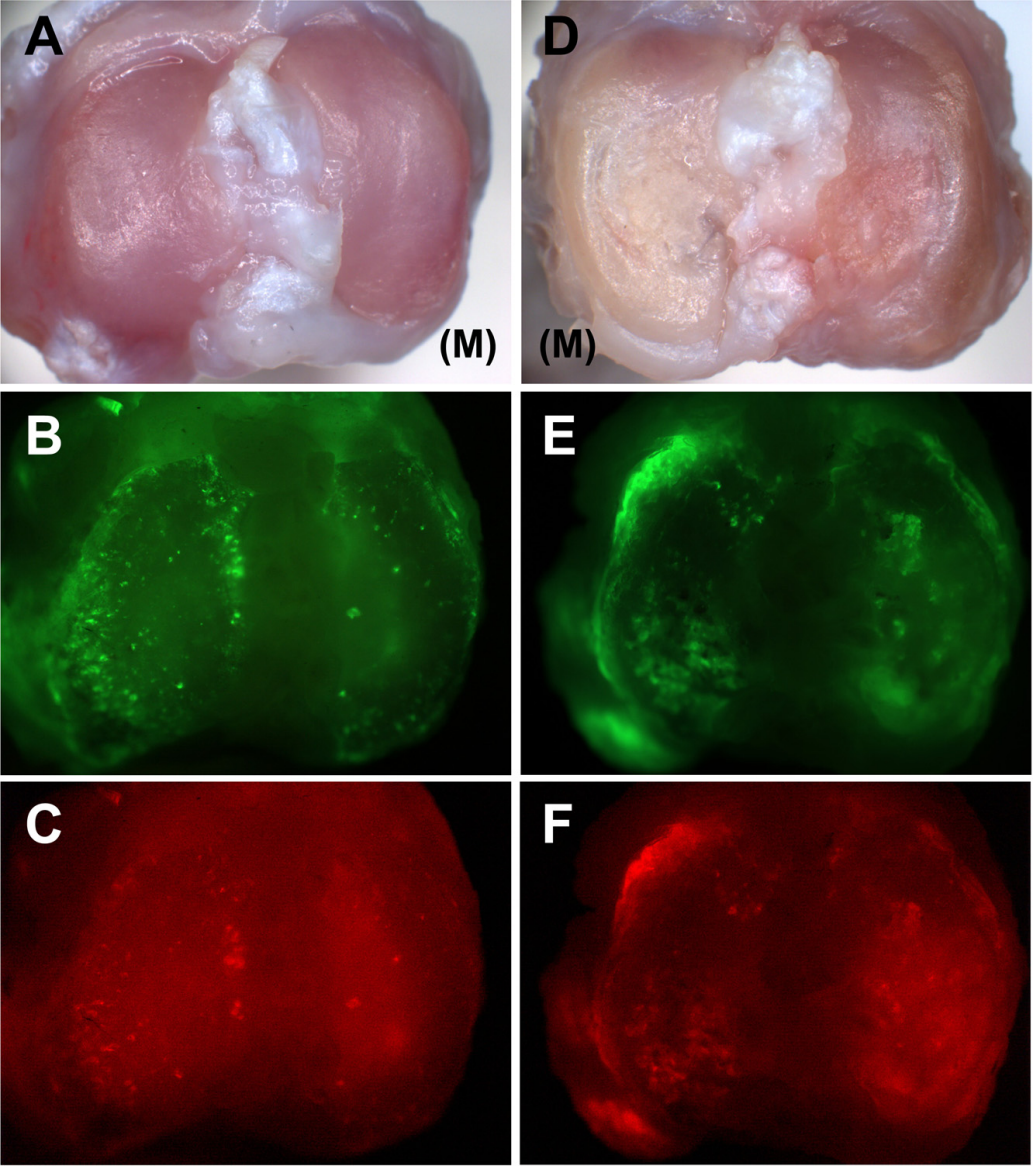
**Macroscopic images of a control tibia (A-C) and of a MIA-injected tibia (D-F), at ten weeks post injection**. The control tibia (A) had no cartilage lesions on the medial compartment (M) and lateral compartment of the tibial plateau, whereas the MIA-injected tibia (D) had severe cartilage lesions on the medial tibial plateau (M). Fluorochrome images of the MIA-injected tibia showed accumulation of calcein and xylenol orange along the margins of the tibial plateau (E, F), suggesting osteophyte formation whereas there was no accumulation of fluorochrome labels along the margins of control tibia (B, C). MIA, monosodium iodoacetate.

All the rats developed cartilage lesions in the tibia of MIA-injected knees. At ten weeks, the tibial cartilage of the MIA-injected knee showed focal lesions with yellow discoloration indicating cartilage degradation (Figure 8A, D). This was more prominent in the central region of the medial tibial plateau, whereas cartilage degradation remained at a moderate stage on the lateral side, giving a total joint grade of 3.16 ± 0.75 (mean ± SD). Macroscopically, the control tibia showed no cartilage lesions in the medial and lateral tibial plateau (joint grade 0). Significant calcein and xylenol orange labels were detected on the subchondral bone and along the margins of the tibial plateau of the MIA-injected knee (Figure 8E, F), indicating active bone formation, whereas no such accumulation of fluorochrome labels was detected in the control tibial plateau (Figure 8B, C).

### Histology

**Figure 9:**
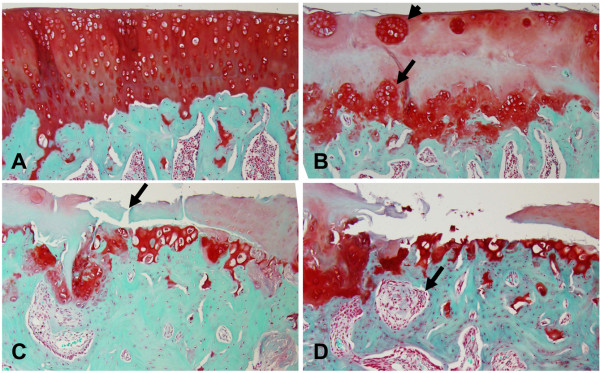
**Coronal sections stained with Safranin O and fast green of a control tibia (A) and of a MIA-injected tibia (B-D) showing the cartilage on the medial tibial plateau, at ten weeks post injection**. The control tibia (A) showed normal healthy cartilage with normally distributed chondrocytes. The MIA-injected tibia (B) showed loss of proteoglycans, loss of viable chondrocytes, chondrocyte proliferation (arrow), and chondrocyte cluster formation of variable sizes (arrowhead). The cartilage showed fibrillation, vertical fissures (C, arrow) and delamination (D). The subchondral trabecular bone architecture was altered with sclerosed bone (C) and the cellular bone marrow was replaced by loosely arranged spindle cells in a fine fibrous stroma (D, arrow). Original magnification ×100. MIA, monosodium iodoacetate.

All the rats showed articular cartilage degradation in the tibia of the MIA-injected knee. The contralateral control tibia had a smooth cartilage surface with evenly distributed chondrocytes, well-oriented and arranged in well-ordered zones (Figure 9A). In the MIA-injected knee, the severity of cartilage damage increased from the posterior margin towards the center of the tibial plateau. Proteoglycan loss was observed with Safranin O, and showed greatly reduced cartilage staining in the center of the tibial plateau. In the areas less affected by cartilage degradation there was partial cartilage damage. The superficial layer was devoid of viable chondrocytes, and disorganized chondrocyte clusters (chondrone) of variable size were observed. There appeared to be proliferation of chondrocytes at the mid and deep cartilage zones that extended into the subchondral bone area (Figure 9B). Vertical fissures were observed at the superficial cartilage zone that extended into the cartilage mid and deep zone (Figure 9C). Coronal sections of the tibial plateau showed cartilage fibrillation and delamination (Figure 9D). These osteoarthritic changes were observed in the medial tibial plateau, whereas the lateral tibial plateau showed relatively mild cartilage changes. The OARSI score of the medial tibial plateau in the MIA-injected knee at 10 weeks was 17 ± 3 (mean ± SD). The control tibia showed no OA activity (OARSI score 0).

In the MIA-injected knee, the subchondral bone was sclerosed and exposed in the medial tibial plateau beneath the areas of cartilage damage. Focally extensive areas of subchondral bone marrow were replaced by loosely arranged spindle cells (fibrotic bone marrow) (Figure 9D). At six and ten weeks, the subchondral plate was breached in focal areas, with cartilage damage and proliferating chondrocytes at ten weeks suggesting fibrocartilage repair (Figure 5B). In four out of six rats, subchondral bone cysts were observed below the articular surface. These cysts were present in the sites of severe bone resorption causing bone marrow lesions. The subchondral bone cysts were surrounded by sclerotic bone with fibrous tissue often containing debris of necrotic bone trabeculae. Some trabeculae adjacent to the sites of bone resorption were lined by a row of large reactive osteoblasts (Figure 5D). Marginal tibial osteophytes were observed in all the rats at ten weeks post-MIA injection of the knee. Bone marrow spaces filled with fibrous cells were observed in these osteophytes (Figure 6D).

### Serum CRP levels

The serum CRP levels were not significantly different between the MIA-injected rats and control rats at any time-point. In addition, there was no significant difference between time points for each of the two groups. In the MIA-injected rats, the mean ± SD concentrations were as follows: 204 ± 32 μg/ml, 201 ± 44 μg/ml, 190 ± 50 μg/ml, and 202 ± 7 μg/ml at pre-injection, 2, 6 and 10 weeks, respectively. For the control group the mean ± SD concentrations were as follows: 172 ± 42 μg/ml, 186 ± 24 μg/ml, 181 ± 34 μg/ml, and 212 ± 9 μg/ml at pre-injection, 2, 6 and 10 weeks, respectively.

## Discussion

This study characterized the temporal changes in the tibial subchondral bone architecture in a low-dose MIA-induced OA rat model using *in vivo *micro-CT. The strength of this study is the use of a longitudinal study design, which enabled the quantitative and qualitative assessment of OA-related changes in the tibial subchondral trabecular bone within the same animal over time. This is the first *in vivo *study to examine subchondral trabecular bone changes over time revealing a significantly lower bone volume fraction at two weeks, followed by subchondral bone sclerosis at six and ten weeks, in the low-dose MIA-induced OA rat model.

The initial bone loss observed at two weeks, followed by sclerosis during the progression of OA is consistent with findings in other animal models of OA, such as the anterior cruciate ligament transection (ACLT) in rats [16], the canine model [17] and the collagenase-induced murine model, and to human OA [18]. This initial bone loss observed at two weeks in the low-dose MIA rat model could have been due to transient unloading of the MIA-injected knee. Previously, it had been shown that MIA injection in the rat knee joint leads to concentration related transient changes in hind paw weight distribution [3,19]. In the MIA-injected knee, Tb.N was decreased and Tb.Sp was increased relative to the control tibia at all time points, which suggests an active process of subchondral trabecular bone erosion. Together with the increases in Tb.Th found at weeks six and ten, these findings indicate that subsequent increased bone formation in the MIA-injected knee resulted from subchondral trabecular bone thickening. This strongly suggests a mechanism for bone remodelling/modelling in the subchondral trabecular bone in OA [20].

It should also be noted that over time the tibial subchondral BV, BV/TV, Tb.Th, Tb.N and Pl.Th increased, whereas the Tb.Sp decreased, in both the MIA-injected knee and the control knee (Figure 3). This is in part due to the normal growth of the rats at baseline, reaching skeletal maturity. Similar age-related changes (increases) in volumetric bone mineral density (vBMD) due to the effect of normal animal growth have been reported in the high-dose MIA rat model [21] and ACLT model [22]. However, in our study the use of the contralateral tibia as an internal control eliminates inter-animal variability for age-related changes and weight.

Pathologic subchondral plate changes are recognized as a characteristic feature of OA. Previous studies have looked at the subchondral plate changes in animal models of OA such as dogs, mice, cynomolgus macaques, guinea pigs, and rabbits but not in rat models of OA. Early subchondral plate thinning of the OA knee followed by an increase in thickness has been reported in the ACLT animal models of OA [17,23,24] and collagen induced OA in mice [13,18]. However, in the low-dose MIA-injected OA animal model, we found an increase in the subchondral plate thickness in the medial tibial plateau at two and six weeks post injection. Such an early increase in the subchondral plate thickness has been previously reported in other animal models of OA such as rabbits [25] and guinea pigs [26], and in studies of hand and knee OA patients [27]. This early increase in subchondral plate thickness observed in the MIA-injected knee could be due to altered loading of the affected knee joint. The thickened subchondral bone could also be associated with progression of cartilage degeneration. In our study, at ten weeks we did not observe the expected increase in the tibial subchondral plate thickness in the MIA-injected knee compared to the control. This could be due to the tibial subchondral plate breach observed in the MIA-injected knee. The increased porosity of the tibial subchondral plate in the MIA-injected knee compared to the control is in line with previous literature [23,24]. This increase in porosity is believed to play an important role in the communication between bone and cartilage during the pathogenesis of OA.

In the present study, the involvement of the subchondral trabecular bone structure after induction of OA using low-dose MIA is clearly shown in a temporal sequence. The two, six and ten week time points were chosen to characterize early, intermediate and advanced OA-related changes in the bone. Because previous studies using the high-dose MIA rat model have reported the involvement of subchondral bone as early as two weeks after injection [21], two weeks was chosen as the early time point in this study. A minimum of four weeks interval is recommended between scans [22], hence, six and ten weeks were chosen as subsequent time points. Given the paired study design, where the contralateral knee was used as an internal control, we prioritized the capture of early subchondral bone changes at two weeks over acquiring baseline data.

Qualitative analysis of micro-CT images revealed subchondral trabecular bone sclerosis at six and ten weeks post-MIA injection in the medial tibial plateau (Figure 4). This thickening of the subchondral trabecular bone was detected by the significant increases in trabecular thickness at six and ten weeks. Subchondral trabecular bone loss together with subchondral bone sclerosis suggests increased osteoclastic and osteoblastic activity that leads to altered subchondral bone remodelling [28].

In our study, micro-CT imaging revealed subchondral plate breach at six and ten weeks post-MIA injection. This was observed in areas of cartilage damage in all the rats (Figure 5A, B). In the weight bearing regions of the medial tibial plateau where the cartilage damage was severe, microfractures were observed in the subchondral plate that exposed the subchondral bone marrow to the joint cavity. This could potentially allow synovial fluid to enter the subchondral bone that triggers cystic cavity formation [29]. Another study suggests that the increased pressure between the delaminated joint surface leads to joint erosion, with synovial breach as a secondary event [30]. A recent study of human OA suggests that cysts may develop in pre-existing bone marrow lesions [31]. The subchondral plate breach also suggests fibrocartilage repair, which could be seen as a discontinuity of the articular bony plate [14]. Januz *et al. *have previously reported subchondral plate breach and its association with subchondral bone sclerosis and cyst formation in the MIA rat model [4]. In our study, 67% of the rats showed subchondral bone cysts in areas of bone resorption (Figure 5C, D).

Another hallmark feature of OA is the presence of osteophytes. In our study, marginal osteophytes were observed from micro-CT images in all the rats at six and ten weeks post-MIA injection. The neochondrogenesis of mesenchymal stem cells in the periosteum and synovial lining has been suggested to be the precursor for osteophyte formation [32]. It is still unclear if osteophytes are simply a pathological phenomenon or a functional adaptation in OA. Macroscopic UV illumination of the MIA-injected knee indicated accumulation of fluorochrome labels along the tibial joint margins and beneath the articular cartilage, demonstrating active osteophyte and subchondral bone formation in all the rats (Figure 8). Such calcein label accumulation was reported previously in an ACLT model for OA in rats [16]. Further histomorphometric analysis of fluorochrome double labels is required to elucidate the rate of bone turnover in tibial subchondral trabecular bone in the MIA-injected knee and control knee.

In the present study, we documented histological changes in the cartilage of the medial tibial plateau at ten weeks post-MIA injection, including loss of proteoglycans, chondrocyte necrosis, chondrocyte proliferation, chondrone formation, cartilage fibrillation and delamination (Figure 9). Similar cartilage changes have been reported in other animal models of OA [33,34]. In this study, articular cartilage damage was analyzed only at the end of the study (ten weeks). Analysis of cartilage changes at two and six weeks would have provided a longitudinal correlation between cartilage loss and subchondral bone changes. However, the main aim of this study was to track the changes in subchondral trabecular bone architecture over time. Moreover, we used a longitudinal study design that allows monitoring of bone changes over time in the same animal, and thus minimizes the number of rats required compared to a cross-sectional study design [5,6].

To our knowledge, this is the first study to show serum levels of CRP in the low-dose MIA induced OA in rats. CRP is an acute phase protein and is a useful non-specific marker of inflammatory diseases [35]. In our study, we have shown that the serum CRP levels of MIA-injected rats do not differ significantly between the baseline and subsequent time points. In addition, there was no significant difference in CRP levels between the low-dose (0.2 mg) MIA-injected rats and control rats at any time point. This demonstrates that a single intra-articular injection of low-dose MIA does not induce marked systemic inflammation in this animal model of OA. Some studies indicated that in human OA the extent and severity of OA is associated with low-grade systemic inflammation with elevated serum CRP levels [36]. However, more recently, the Rotterdam Study-I, the largest cohort study to date, concluded that acute systemic inflammation is not present in OA and future studies should focus on the local inflammation process involved in OA [37].

It is hypothesized that in OA, cartilage matrix breakdown causes synovial inflammation and subsequent changes in the knee joint [38]. In the low-dose MIA rat model, the cartilage breakdown products could lead to mild local inflammation and synovitis in the knee joint that promotes subchondral bone changes. In our study, we were unable to observe for the presence of infiltrated local inflammatory cells as histology was performed only on the tibia and not the whole knee joint. Previous studies using the high-dose MIA rat model have reported the presence of acute inflammation characterized by expansion of the synovial membrane with infiltrating macrophages, neutrophils and plasma cells in the rat's knee joint one day after injection which resolved by day seven [19,28]. In a more recent study, increased activation of macrophages in the knee joint was reported in a high-dose MIA (1 mg) rat model up to four weeks after injection [39].

Although the low-dose MIA model mimics articular cartilage and subchondral bone pathology of human OA, like any other animal model of OA it does not fully mirror human OA. Barve *et al. *(2007) reported that 0.2 mg MIA induced a consistent matrix loss of approximately 35%, similar to the observed range of human OA samples. However, they found that the transcriptional similarity between the MIA rat and human OA cartilage was modest [40]. This modest similarity between rat and human cartilage could have been due to a difference in sampling site, sampling of lesion and non-lesion containing cartilage, stages of OA, and contamination of cartilage by underlying bone. Moreover, this study did not look at the protein expression or post translational modification that may demonstrate greater similarity between MIA rat and human OA cartilage. Other factors such as species variability, tissue heterogeneity and differences in underlying molecular mechanisms need to be considered. The choice of an animal model of OA mainly depends on the purpose of a study. The main purpose of our study was to characterize a non-trauma animal model that mimics tissue-level features of human OA, which could be used to study the effect of disease modifying OA drugs on cartilage and subchondral bone.

## Conclusions

In summary, the changes in cartilage and subchondral trabecular bone structure observed in the low-dose MIA rat model reveal important pathologic features of human OA and the temporal progression of the disease in the tibial subchondral bone. The low-dose MIA induced OA rat model mimics the human disease condition and clearly demonstrates disease progression in the tibial subchondral bone in a timely manner. This animal model is suitable to study the effect of suitable therapeutic drugs on cartilage and subchondral bone. *In vivo *micro-CT enabled the temporal characterization of the changes in subchondral bone architecture both quantitatively and qualitatively in 3D. The changes in subchondral trabecular bone structure were found to be consistent with disease progression, as confirmed by end-stage histology. *In vivo *micro-CT is a non-destructive imaging technique that can be used in future studies to track changes in subchondral bone structure in drug intervention studies designed to slow the progression of OA in a non-trauma low-dose MIA induced OA rat model.

## Abbreviations

ACLT: anterior cruciate ligament transection; ANOVA: analysis of variance; BV: bone volume; BV/TV: bone volume fraction; CRP: C-reactive protein; DMOAD: disease modifying osteoarthritis drugs; MIA: monosodium iodoacetate; Micro-CT: micro computed tomography; OA: osteoarthritis; OARSI: Osteoarthritis Research Society International; Pl.Por: plate porosity; Pl.Th: plate thickness; ROI: region of interest; Tb.N: trabecular number; Tb.Sp: trabecular separation; Tb.Th: trabecular thickness; vBMD: volumetric bone mineral density; VOI: volume of interest; 3D: three dimensional.

## Competing interests

The authors declare that they have no competing interests.

## Authors' contributions

GM undertook all data acquisition and had full access to all the data in the study; GM, EP, JSK, IHP, and NLF participated in the study design and had final responsibility for the decision to submit for publication; GM and EP participated in the data analysis; GM and JMH carried out immunoassay and CRP data analysis; GM, EP, JSK, JMH, IHP, and NLF participated in the interpretation of the study data. All authors have participated in the preparation of the manuscript and have seen and approved the final version.
